# Synthesis and characterizations of o-nitrochitosan based biopolymer electrolyte for electrochemical devices

**DOI:** 10.1371/journal.pone.0212066

**Published:** 2019-02-15

**Authors:** Noriah Abdul Rahman, Sharina Abu Hanifah, Nadhratun Naiim Mobarak, Mohd Sukor Su’ait, Azizan Ahmad, Loh Kee Shyuan, Lee Tian Khoon

**Affiliations:** 1 Faculty of Science and Technology, Universiti Kebangsaan Malaysia, Bangi, Selangor Darul Ehsan, Malaysia; 2 Polymer Research Center (PORCE), Faculty of Science and Technology, Universiti Kebangsaan Malaysia, Bangi, Selangor Darul Ehsan, Malaysia; 3 Solar Energy Research Institute (SERI), Universiti Kebangsaan Malaysia, Bangi, Selangor Darul Ehsan, Malaysia; 4 Fuel Cell Institute (FCI), Universiti Kebangsaan Malaysia, Bangi, Selangor Darul Ehsan Malaysia; East China Normal University, CHINA

## Abstract

For the past decade, much attention was focused on polysaccharide natural resources for various purposes. Throughout the works, several efforts were reported to prepare new function of chitosan by chemical modifications for renewable energy, such as fuel cell application. This paper focuses on synthesis of the chitosan derivative, namely, O-nitrochitosan which was synthesized at various compositions of sodium hydroxide and reacted with nitric acid fume. Its potential as biopolymer electrolytes was studied. The substitution of nitro group was analyzed by using Attenuated Total Reflectance Fourier Transform Infra-Red (ATR-FTIR) analysis, Nuclear Magnetic Resonance (NMR) and Elemental Analysis (CHNS). The structure was characterized by X-ray Diffraction (XRD) and its thermal properties were examined by using differential scanning calorimetry (DSC) and thermal gravimetric analysis (TGA). Whereas, the ionic conductivity of the samples was analyzed by electrochemical impedance spectroscopy (EIS). From the IR spectrum results, the nitro group peaks of O-nitrochitosan, positioned at 1646 and 1355 cm^-1^, were clearly seen for all pH media. At pH 6, O-nitrochitosan exhibited the highest degree of substitution at 0.74 when analyzed by CHNS analysis and NMR further proved that C-6 of glucosamine ring was shifted to the higher field. However, the thermal stability and glass transition temperatures were decreased with acidic condition. The highest ionic conductivity of O-nitrochitosan was obtained at ~10^−6^ cm^-1^. Overall, the electrochemical property of new O-nitrochitosan showed a good improvement as compared to chitosan and other chitosan derivatives. Hence, O-nitrochitosan is a promising biopolymer electrolyte and has the potential to be applied in electrochemical devices.

## Introduction

Chitosan is a linear polysaccharide which has *D*-glucosamine units and *N*-acetyl-*D*-glucosamine units linked by β (1 → 4) [1]. Chitosan can be obtained from the partial deacetylation process of chitin through alkaline treatment by using sodium hydroxide. Chitin can be easily found in marine resources, in the shells of crustaceans, such as crabs, shrimps, the cuticles of insects, and the cell walls of fungi [2–4]. Chitin and chitosan are classified as natural polysaccharide and renewable resources which are currently being intensively explored due to their excellent properties, such as biocompatible, biodegradability, non-toxicity and adsorption properties. Therefore, chitosan is used for many applications, such as pharmaceutical drug delivery system [5], water treatment by removing heavy metal [6,7] and fuel cell [8].

However, chitosan exhibits low conductivity at room temperature between 10^−10^ and 10^−9^ S cm^-1^[9]. In the previous study, the conductivity of chitosan can be improved by adding lithium and ammonium-based salts [10,11], in which complexation takes place through the salt-polymer interaction. Nonetheless, its conductivity is influenced and dependent on the number of free conducting species and degree of dissociation of the salt. Alternatively, the electrochemical properties of the chitosan can be ameliorated through chemical modification. Chitosan can be chemically modified at its free amino group, NH_2_ at the C-2 position or nonspecific reactions of—OH groups at the C-3 and C-6 positions. These two functional groups also have lone pair of electrons that are suitable for the preparation of solid polymer electrolytes [8]. These functional groups, which are also introduced during chemical modification, will enhance the interaction and complexation with the salt [12]. For the past decades, many chitosan derivatives were synthesized with various methods, such as carboxymethyl chitosan through alkylation [13], phosphorylation [14], quaternary salt formation [15] and sulfonation [16]. Notwithstanding, the exploration and modification of chitosan to its derivatives specifically for polymer electrolyte used in electrochemical devices are still considered new. However, the existence of nitro group in O-nitrochitosan may have high potential to be used as anion exchange membranes. The O-nitrochitosan is expected to have low methanol permeability and better conductivity due to higher water content to be applied in PEMFC.

A few types of chitosan derivatives were explored as solid biopolymer electrolyte (SBE). For example, N-phthaloylchitosan (PhCh) was synthesized by reacting chitosan with phthalic anhydride in dimethylformamide (DMF). Carboxymethyl chitosan was also synthesized, by using monochloroacetic acid and natrium hydroxide. A list of chitosan derivatives and conductivity are tabulated in Table 1, according to the previous studies.

**Table 1:** Different blank chitosan derivatives with their conductivity values.

Chitosan derivatives film	Conductivity (S cm^-1^)	Authors
*N*-methylene phosphonic chitosan (NMPC)	3.6 × 10^−6^	Liew et al. [17]
*N*-propylsulfonic acid chitosan (SC)	4.4 ×10^−7^	Cardoso et al. [12]
Carboxymethyl chitosan (CMCS)	4.0 × 10^−7^	Mobarak et al. [13]
*N*-phthaloylchitosan (PhCh)	5.0 × 10^−9^	Aziz et al. [18]

**Fig 1:**
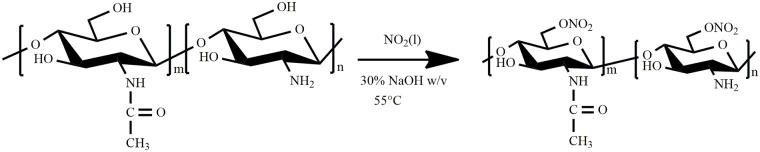
The synthesis scheme of O-nitrochitosan.

From the best of our knowledge, *O*-nitrochitosan has not been used for electrochemical device or biopolymer electrolyte. O-nitrochitosan molecule structure has the potential to provide sufficient oxygen to form complexation with cation which will take place when an electric field is applied during conduction. The most similar structure to *O*-nitrochitosan is carboxymethyl chitosan (CMCS). However, carbon was replaced with nitrogen that is more electronegative. Comparison between four functional groups, NO_2_^-^ > PO_3_^3-^> COO^-^ > SO_3_^2-^ were discussed. It was reported that nitro group had the highest electronegativity thus produced the highest conductivity value, 5.22 ×10^−6^ S cm^-1^ for the blank system compared to PO_3_^-3^, COO^-^ and SO_3_^-2^ which their conductivity values were 3.6 ×10^−6^ S cm^-1^ [17], 4.0 ×10^−7^ S cm^-1^ [13] and 5.0 × 10^−9^ S cm^-1^ [18], respectively. In this work, O-nitrochitosan which was synthesized in different ratios of sodium hydroxide was studied. The synthesis route of O-nitrochitosan is illustrated in Fig 1. Further study was done to determine the potential of O-nitrochitosan as biopolymer electrolyte. This biopolymer membrane is expected to have higher conductivity as compared to its predecessor, the chitosan.

## Experiment

### Materials

Chitosan, medium molecular weight (190,000–310,000) was commercially obtained from Sigma Aldrich. Sodium hydroxide and organic solvent of isopropanol (99.9%), methanol (95%), ethanol (95%) supplied by Systerm were used as solvents. All chemicals were used without further purification. Nitric acid Fume was synthesized in-house by distillation of nitric acid and sulfuric acid at 80 °C for 2 h.

### Preparation of O-nitrochitosan powder

The O-nitrochitosan powder was prepared according to the previous work, but with a modification of NaOH volume used [19]. The O-nitrochitosan powder was prepared by adding an amount of chitosan in isopropanol solution and stirred. Thereafter, the solution was introduced with 10%, 20% and 30% of sodium hydroxide, respectively. The pH medium of the chitosan solution was measured by using a pH meter. Then, the solution was stirred for 1 h before the nitric acid fume was added dropwise to the mixture at 55°C for 4 h. Next, O-nitrochitosan was purified via precipitation in methanol and filtered. Afterward, O-nitrochitosan was washed with ethanol for four times. The product was filtered and oven dried at 100 °C. The pH values were only stated as the effect after a certain volume of sodium hydroxide was added before reacting with nitric acid (Table 2).

**Table 2:** The volume of NaOH added to each sample and the pH value.

Sample labels	NaOH, w/v (%)	pH
Chitosan	-	-
*O*-nitrochitosan pH 1	10%	1
*O*-nitrochitosan pH 2	20%	2
*O*-nitrochitosan pH 6	30%	6

### Preparation of film samples

The O-nitrochitosan films were prepared via solution casting technique. An appropriate amount of O-nitrochitosan powder was dissolved in 1% (v/v) aqueous acetic acid solution. The mixture was stirred for 24 h until all O-nitrochitosan powder was completely dissolved and formed a homogenous solution. It was then cast into the petri dish and left to form films, approximately for 72 h in the fume hood. The films were kept in the desiccator for continuous drying.

### Characterizations

#### Attenuated Total Reflection- Fourier transform infrared spectroscopy (ATR-FTIR)

ATR-FTIR analysis was carried out by using Perkin Elmer Spectrum 400 FTIR/FT-NIR spectrophotometer. These spectra were collected in the range of 4,000 cm^-1^ to 650 cm^-1^ with a resolution of 2 cm^-1^. This analysis was conducted to observe changes or shifts in wavelength after comparison between chitosan and chitosan derivative that refers to O-nitrochitosan.

#### Nuclear Magnetic Resonance (NMR)

The changes in the chemical structures of chitosan and O-nitrochitosan were recorded by using ^1^H and ^13^C NMR from Bruker AVANCE III 600 MHz spectrometer. The chitosan and its derivatives were dissolved in the mixture of D_2_O and CD_3_COOD.

#### Elemental analysis (CHNS)

The sample properties were further studied by elemental analysis, CHNS by using TruSpec Micro model. The results can be used to determine the degree of substitution (DS) by using Eq (1) with some modification from [20] Gan et al. 2017.

$$\text{DS}=\frac{\left(169\times \%\text{N}\right)}{\left[(14\times 100)-46\times \%\text{N}\right]}$$ (1)

Where, N (%) is the nitrogen content of modified O-nitrochitosan, 169 is the molecular weight of anhydroglucose unit (AGU), 14 is the molecular mass of nitrogen atom and 46 is the net increment in the AGU for every substituted nitro group.

#### X-ray diffraction (XRD)

The X-ray diffraction was employed to determine the crystallinity of prepared samples. XRD was carried out by using XRD D8 Advance Bruker. The data were collected at room temperature from the range diffraction angle 2θ of 2° to 80° and at the scan rate of 0.05° s^-1^. Samples in powder and film formed were used in this analysis.

#### Thermal analysis

Thermal properties of O-nitrochitosan powder were determined by differential scanning calorimetry (DSC) supplied by Mettler Toledo Model 822c and thermal gravimetric analysis (TGA) was carried out by using Mettler Toledo model TGA/SDTA 851. Glass transition temperature (T_g_) from DSC analysis of the chitosan and its derivatives were measured at a heating scan rate of 10 °C/min from 30 °C to 200 °C under nitrogen gas flow. TGA was carried out to investigate the chitosan and O-nitrochitosan decomposition. The samples were heated from 25 °C to 600 °C under argon flow at a heating rate of 10 °C/min.

#### Electrochemical impedance spectroscopy (EIS)

The value of conductivity of the film samples was characterized by EIS through VersaSTAT (Versastudio software) in the frequency range of 0.1 Hz to10 MHz with 100 mV amplitude. The films were cut into a suitable size and set up in the form of a sandwich between the stainless steel ion-blocking electrodes of conductivity cell with a surface contact area of 1.732 cm^2^ and connected to a computer [21]. The bulk resistance (R_b_) was determined from the equivalent circuit analysis by using the Zview analyzer software. The impedance data were processed by Zview in a complex impedance plot, where the imaginary part, Z_i_ (Z’) was plotted against its real part Z_r_ (Z”). The electrical conductivity (σ) of the films was calculated from Eq (2):

$$\sigma =\frac{t}{R_{b}A}$$ (2)

Where, t is the film thickness (cm) and A is the film-electrode contact (cm^2^). The analysis was performed at room temperature [22].

### Results and discussion

#### ATR-FTIR analysis of O-nitrochitosan powder

**Fig 2:**
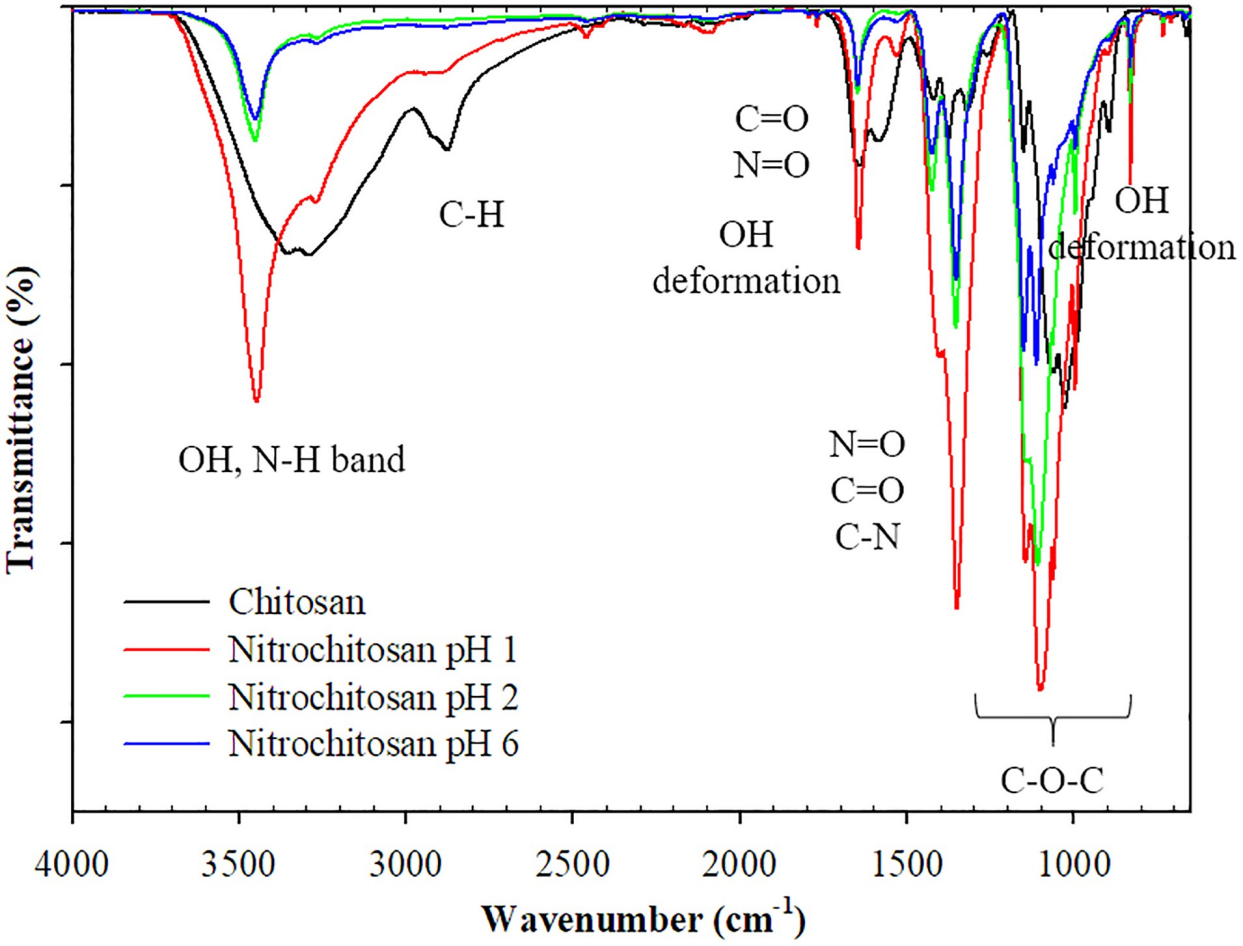
ATR-FTIR spectra of chitosan and its derivatives (powdered form) in acidic medium. The data is available in S1 Fig.

The chemical properties of O-nitrochitosan powder were characterized by using ATR-FTIR, ^1^H NMR and CHNS analyses. Fig 2 depicts the ATR-FTIR spectra for chitosan and its derivatives in powdered form. ATR-FTIR spectrum shows all the main six peaks for chitosan. First, a strong and broad stretching peak band of O-H can be seen overlapping with N-H peak at 3328 cm^-1^ as reported by Samantha et al. [23]. Second, peak axial stretching of C-H is depicted at 2872 cm^-1^. Three types of amide groups of chitosan, which were identified at 1644 cm^-1^, 1569 cm^-1^ and 1320 cm^-1^, corresponded to C = O stretching (amide I), N-H bending (amide II), and C-N stretching (amide III), respectively [23,24]. Finally, the peaks in the range of 1151–897 cm^-1^ shifted to the left (1065–993 cm^-1^), corresponding to symmetric and asymmetric stretching vibrations of C-O and C-O-C polysaccharide skeleton [25].

After modification, a strong and wide band of O-H that overlapped with N-H band (3349 cm^-1^) in chitosan became sharper and the N-H peak shifted to the left to 3453 cm^-1^. This proved that the quantity of O-H stretched decreased as nitro group substituted on one of three hydroxyl groups in chitosan. C-H peak also shifted to the left to 3262 cm^-1^. The introduction of nitro group to the chitosan resulted in two new sharp peaks, which appeared at 1,646 cm^-1^ and 1,355 cm^-1^. The sharp peak at 1646 cm^-1^ was the combination of three peaks, namely C = O stretching, asymmetric NO_2_ stretching and OH deformation vibrations which associated with amide I group [7]. On the other hand, the peak at 1355 cm^-1^ was the combination peak of symmetry NO_2_, C = O stretching, CH_3_ deformation and C-N stretching vibration. The other OH deformation peak can be seen at 827 cm^-1^, as being reported by the previous study [7].

#### ^1^H-NMR analysis for O-nitrochitosan powder

**Fig 3:**
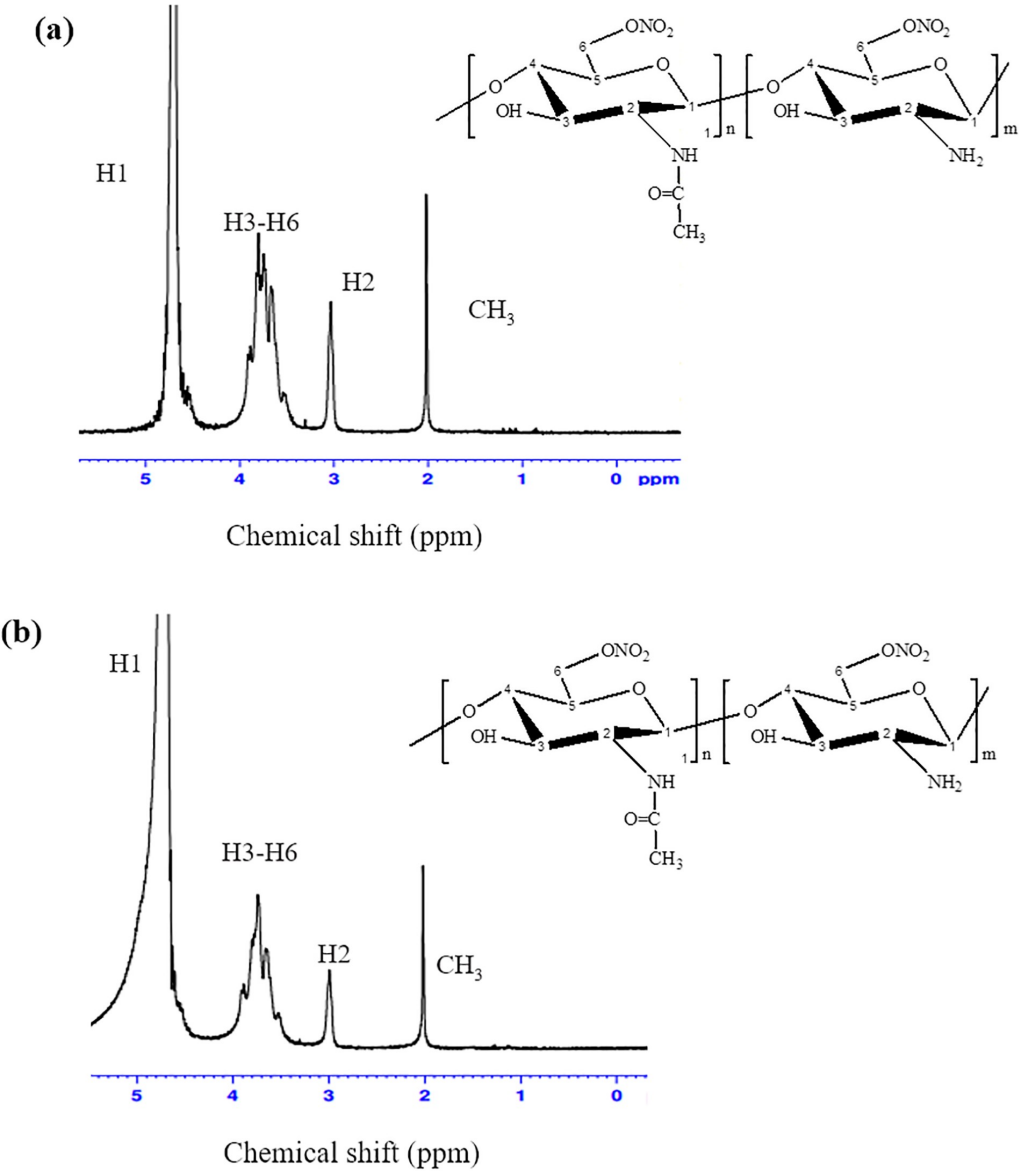
^1^H NMR spectrum of O-nitrochitosan at (a) pH 1 and (b) pH 6 in D_2_O. The inset shows the repeating unit of chitosan (m < n).

Fig 3 depicted the ^1^H NMR spectrum of *O*-nitrochitosan at various pH media in D_2_O. The proton assignments of O-nitrochitosan pH 1 were as follows (ppm): ^1^H NMR (D_2_O):2.01 (CH_3_, acetamido group of chitosan), 4.60 (H1), 3.03 (H2), 3.82 (H3), 3.89 (H4), 3.67 (H5), 3.91, 3.77 (H6). The proton assignments of O-nitrochitosan pH 6 were as follows (ppm): ^1^H NMR (D_2_O):2.01 (CH_3_, acetamido group of chitosan), 4.70 (H1), 3.00 (H2), 3.77 (H3), 3.89 (H4), 3.65 (H5), 3.91 (H6). The proton assignments of chitosan were as follows (ppm): 1.96 (CH_3_, acetamido group of chitosan), 4.70 (H1), 3.06 (H2), 3.75 (H3), 3.80 (H4), 3.63 (H5), 3.80, 3.65 (H6) [19]. The proton at C-6 of glucosamine ring of O-nitrochitosan, at both pH 1 and pH 6, were higher than the proton in chitosan due to the substitution of nitro group at C6 in both materials; thus, the electronegativity of the proton shifted to the higher field.

#### Elemental analysis (CHNS) analysis for O-nitrochitosan powder

Table 3 shows the percentage of carbon, oxygen, nitrogen and degree of substitution (DS) of chitosan as well as *O*-nitrochitosan. Elemental analysis is used to determine the degree of substitution that will take place after some modification is completed. Through this analysis, we can estimate the number of nitro group that will substitute the two hydroxyl groups in the chitosan structure after modification with nitration process in different percentage of NaOH media. The DS of nitro can be calculated from Eq (1). The DS of *O*-nitrochitosan were obtained at 1.22, 1.12 and 1.28. After the modification, the nitrogen and oxygen content were increased. This can be seen when the percentage of nitrogen and oxygen in the derivatives chitosan increased compared to the chitosan. Subsequently, the increase of nitro group has enchanced the conductivity of chitosan derivatives, which can be seen in latter part (proton conductivity section). The nitro group which possesses strong electron-withdrawing property will facilitate the coordination with the proton [13]. Thus, the ion mobility will increase.

**Table 3:** Elemental percentage of chitosan and O-nitrochitosan.

Samples	Carbon (%)	Hydrogen (%)	Nitrogen (%)	Oxygen (%)	DS
Chitosan	40.04	5.80	6.30	46.97	-
*O*-nitrochitosan pH 1	19.60	3.68	7.60	69.12	1.22
*O*-nitrochitosan pH 2	18.86	3.68	7.10	70.35	1.12
*O*-nitrochitosan pH 6	26.95	5.14	7.86	60.05	1.28

#### Mechanism of O-nitrochitosan

**Fig 4:**
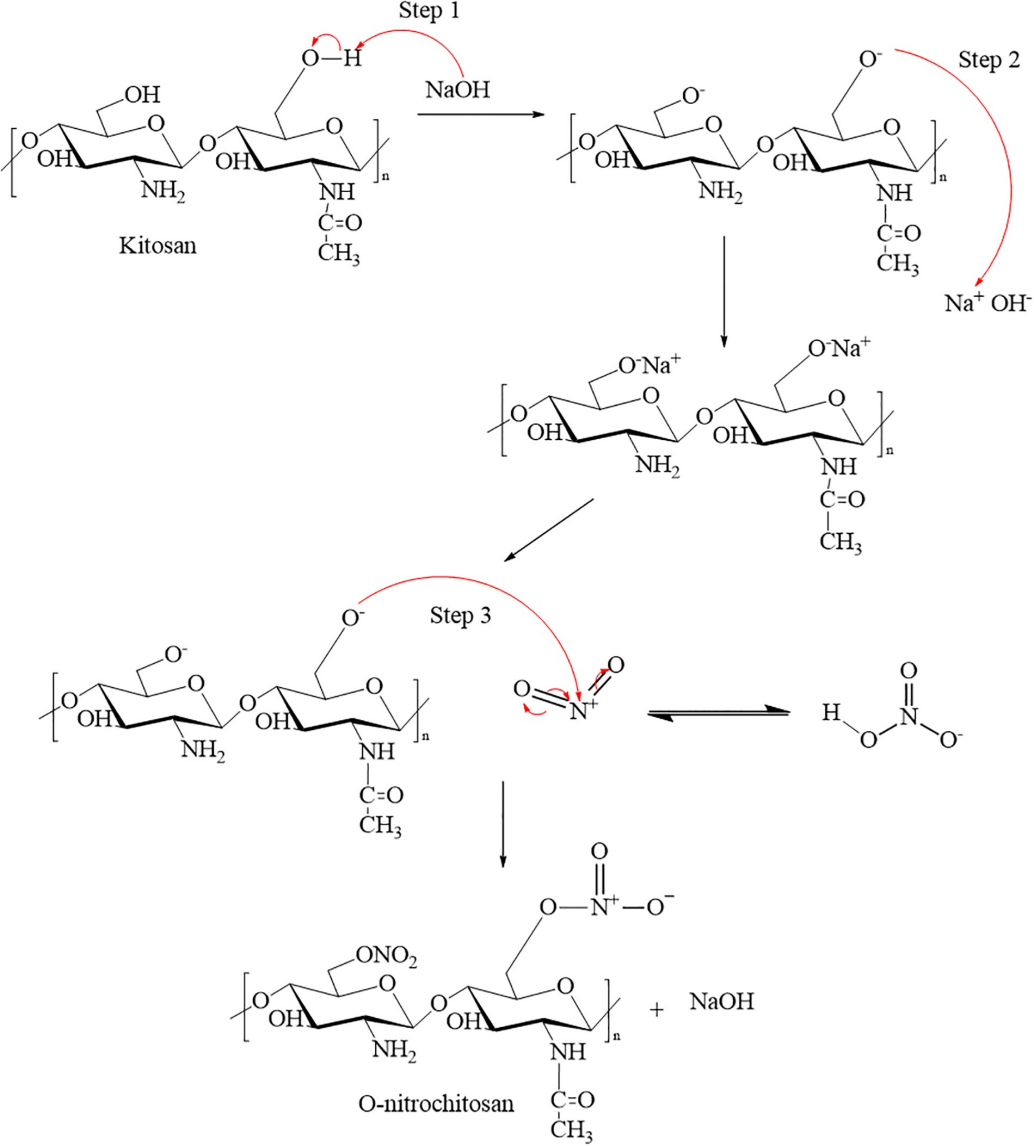
The proposed mechanism from chitosan to O-nitrochitosan.

The mechanism of O-nitrochitosan began in the acidic medium with the base abstracting the proton from the alcohol by using a base as soon as sodium hydroxide was added, as shown in Step 1. In the chitosan structure, the reaction only occurred at three places; C2, C3, and C6. This reaction occurred at C6 because it was easier to react since the C6 position was outside of the chitosan ring. Next, alkoxide will attract the Na^+^ from NaOH (Step 2) to form sodium salt chitosan formation. This formation was temporary. When the sodium left from sodium salt chitosan formation, it will make the alkoxide formation again. Then, the alkoxide will donate its electron to the electrophilic nitronium when the nitric acid fume was added to the reactant and formed O-nitrochitosan, as seen in Step 3. The reaction that took place in the overall reaction was SN_2_ replacement. Fig 4 shows the proposed reaction mechanism of O-nitrochitosan in the acidic medium.

#### XRD analysis of powder

**Fig 5:**
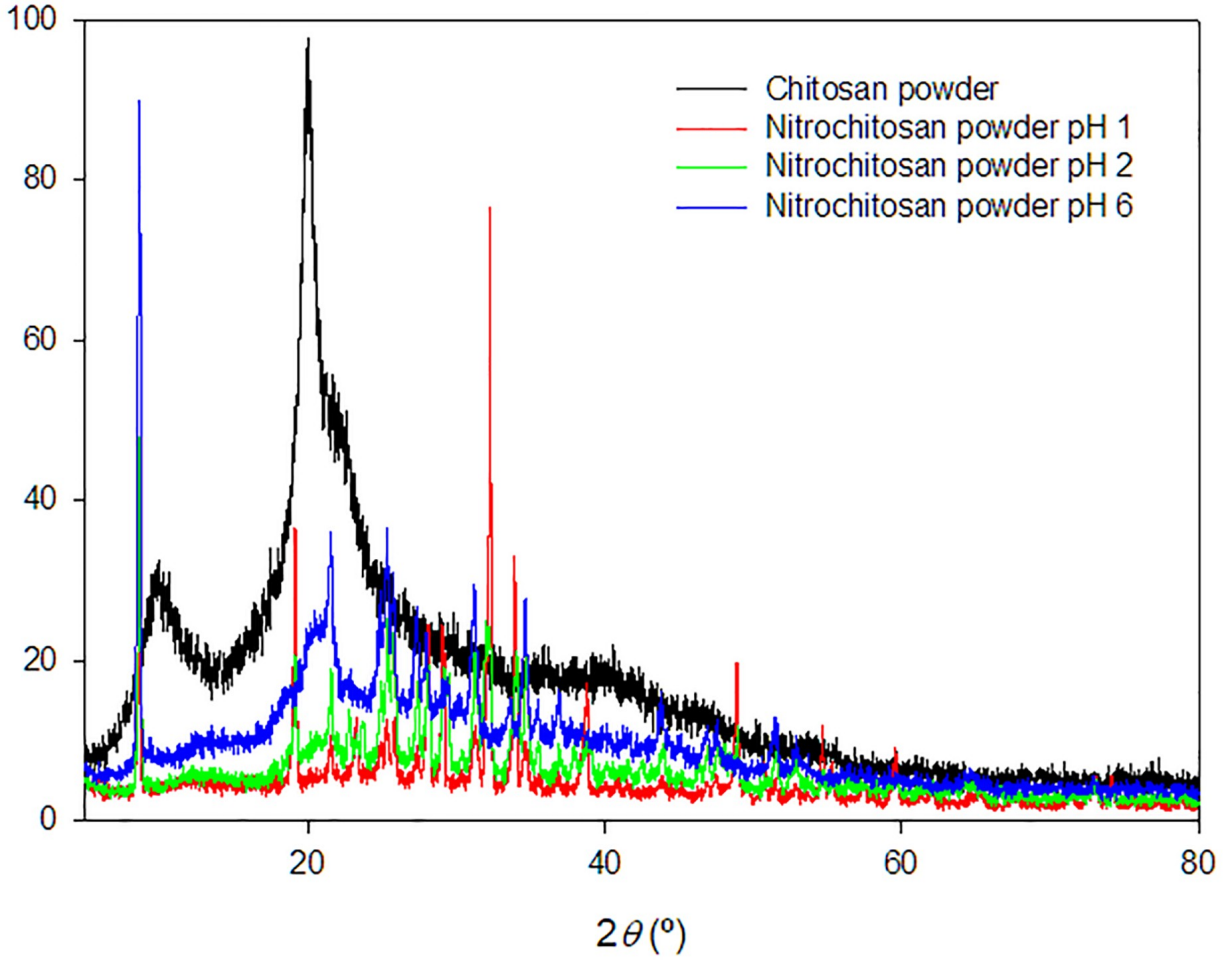
XRD diffractogram of chitosan powder and its derivatives. The data is available in S2 Fig.

XRD diffractogram of chitosan powder and its derivatives are shown in Fig 5. From diffractogram of chitosan powder, the presence of broad peaks, at around 2θ = 9.4 °, 20.0 ° and 28.4°, showed that chitosan exhibited a high degree of crystallinity phase [13,26]. After modification with the addition of nitro group, the intensity at the broad peaks of chitosan derivatives were decreased. However, small and sharp peaks appeared at 8.45 ° and from 20 ° to 50 ° region, which indicated that the crystallinity increased at some parts of chitosan as number of hydrogen bond also increased when it was derived from O-nitrochitosan. These findings were supported by glass transition temperature (*T*_*g*_) of chitosan which was 73 °C, while the chitosan derivatives at different pH media obtained higher *T*_*g*_ from 78°C to 80 °C.

#### Thermal analysis

**Fig 6:**
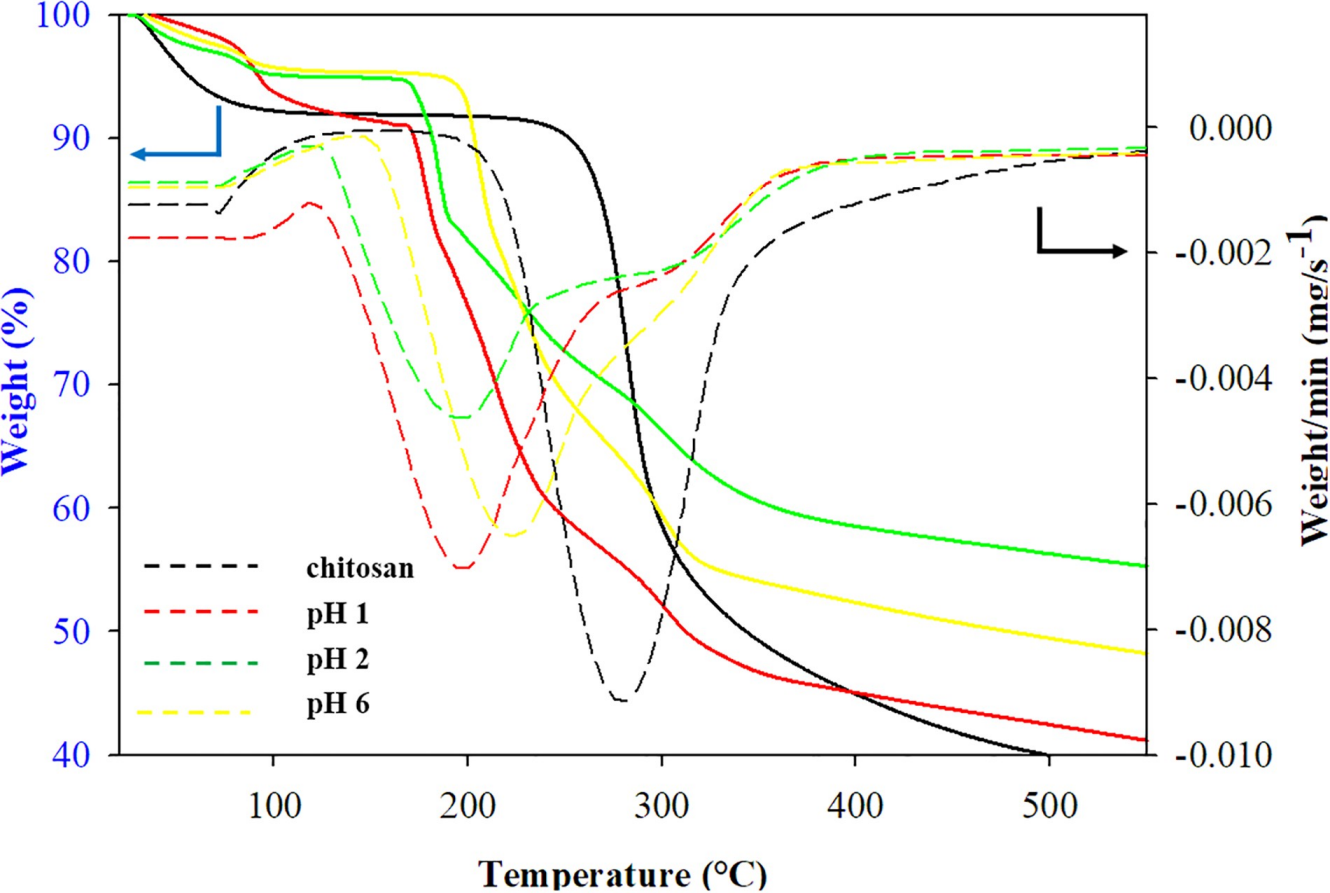
TGA and DTG thermograms of chitosan and its derivatives. The data is available in S3 Fig.

TGA and DTG curves of chitosan and its derivatives are shown in Fig 6 and tabulated in Table 4. As we can see from the thermogram, chitosan has two stages of degradation and O-nitrochitosan has three stages of degradation. For chitosan, the first stage began at 80 °C and up to 100°C with a weight loss of about 4% to 8%, which was due to water loss, which is related to the hydrophilic nature of chitosan [27]. For the second stage of degradation, it began at about 280°C, which contributed from depolymerization and decomposition of the basic unit of polymer polysaccharides. For O-nitrochitosan, the first degradation was referred to the water loss. A weight loss of more than 52% started at about 197 °C, which attributed to the degradation of glycerol and depolymerization in chitosan, was the second stage of degradation [27]. Next, the third stage of degradation happened after 280 °C as we can see from TGA and DTG thermogram, as shoulder peak as referred to the weight loss of the nitro functional group. The introduction of nitro group to the structure of chitosan has shifted the second main peak from 280 °C to 197°C and caused the third stage of degradation. Fig 6 shows the TGA and DTG thermograms.

**Table 4:** Temperature of degradations and number of degradation steps of chitosan powder and its derivatives.

Samples	First stage	Second stage	Third stage	No. of stages
Chitosan	79	280	-	2
O-nitrochitosan pH 1	81	197	300	3
O-nitrochitosan pH 2	79	197	319	3
O-nitrochitosan pH 6	78	224	315	3

**Fig 7:**
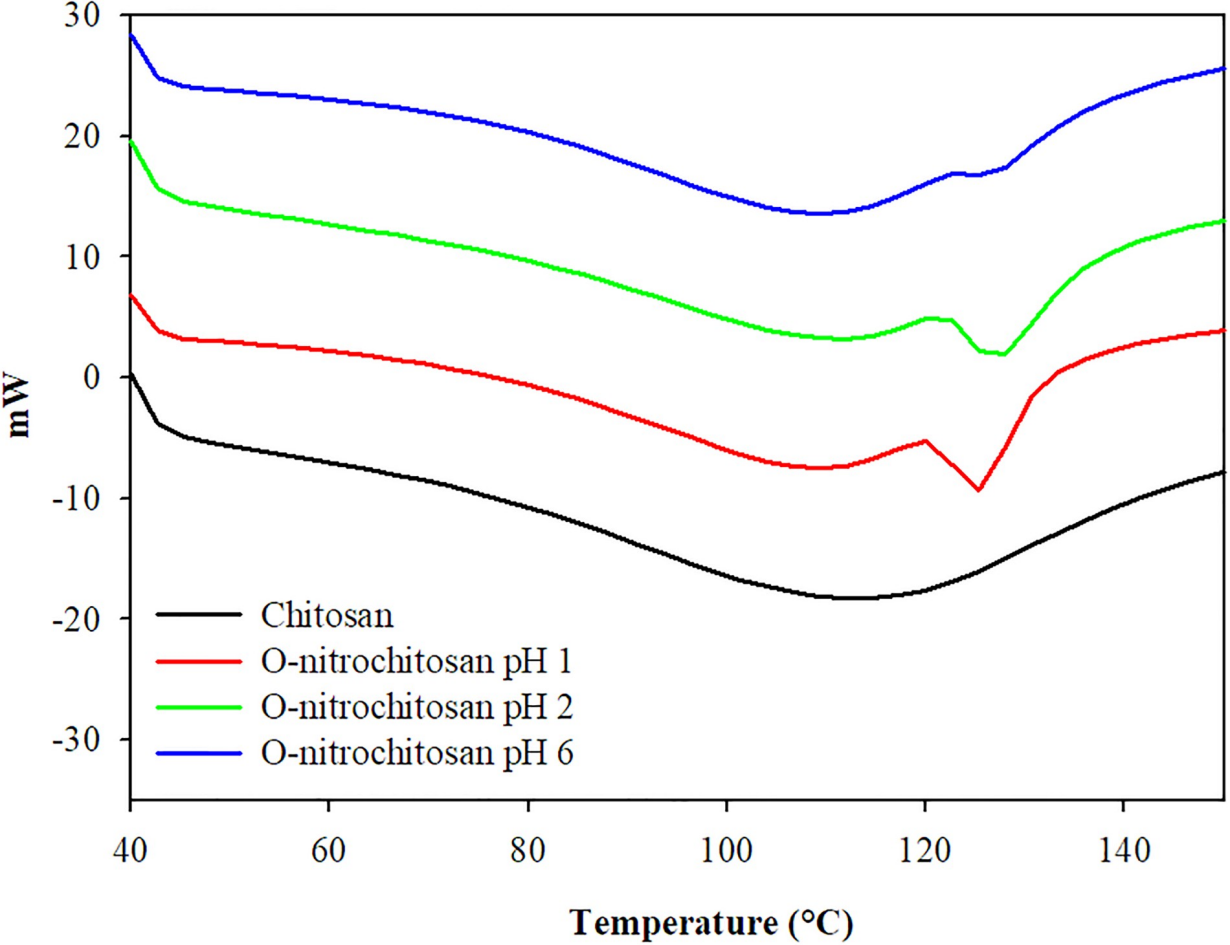
Second run thermal analysis curves for (a) chitosan, O-nitrochitosan pH 1, pH 2 and pH 6. The data is available in S4 Fig.

Fig 7 shows the second run of DSC thermogram of O-nitrochitosan pH 1, pH 2 and pH 6 in the temperature range of 0°C –590 °C. From the DSC thermogram, traces of the chitosan were seen at the first endothermic peak, at 80–100°C, which attributed to absorb moisture [27]. Polysaccharide like chitosan has a strong affinity towards water and their endotherm is normally related to the evaporation of water. These molecules are different in water holding capacity; thus, it will affect the strength of water-polymer interaction. The endothermic values were observed to be higher in chitosan derivatives than chitosan itself. This may correspond to the evaporation of water which reflected the physical and molecular changes during chemical modification [28, 29]. The *T*_g_ temperature for chitosan derivatives was about 120°C to 123°C. Researchers have found that chitosan has a few values of glass transition [30,31]. Chitosan has a lower glass temperature of 73°C than its derivatives, which were around 78°C-80°C. Table 5 shows the glass transition temperature for chitosan powder and its derivatives.

**Table 5:** Glass transition temperature for chitosan powder and its derivative.

Samples	Glass transition (°C)
Chitosan	73
O-nitrochitosan pH 1	78
O-nitrochitosan pH 2	79
O-nitrochitosan pH 6	80

#### Ionic conductivity

**Fig 8:**
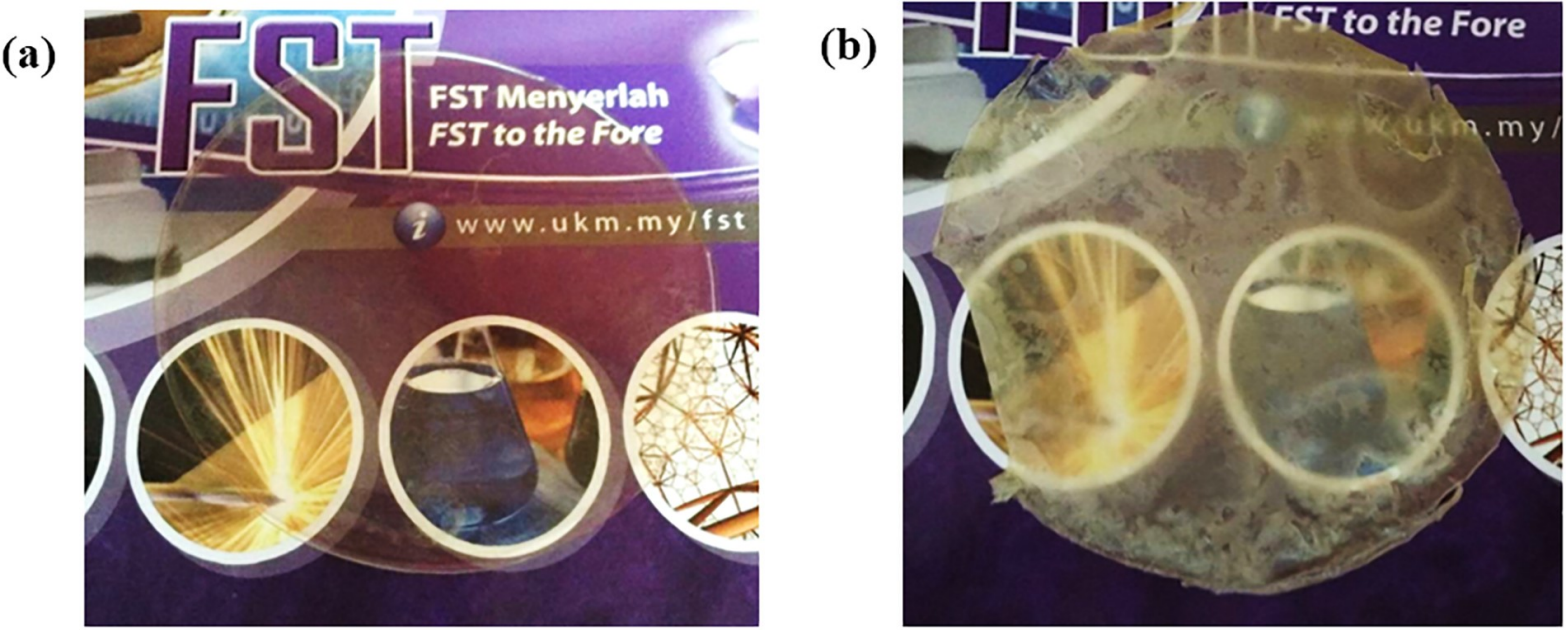
Physical observation of (a) chitosan film and (b) nitrochitosan film.

The potential of O-nitrochitosan as solid biopolymer electrolytes was investigated by determining its ionic conductivity. The nitrochitosan films were yellowish in color, semi-transparent and flexible, as shown in Fig 8, whereas the conductivity results of the films are tabulated in Table 6.

**Table 6:** The conductivity of the nitrochitosan film.

Samples	Bulk resistance (R_b_)	Conductivity (S cm^-1^)
Chitosan	6.40 × 10^6^	8.88 × 10^−10^
O-nitrochitosan pH 1	5.85 × 10^3^	9.02 × 10^−7^
O-nitrochitosan pH 6	8.23 × 10^2^	5.22 × 10^−6^

**Fig 9:**
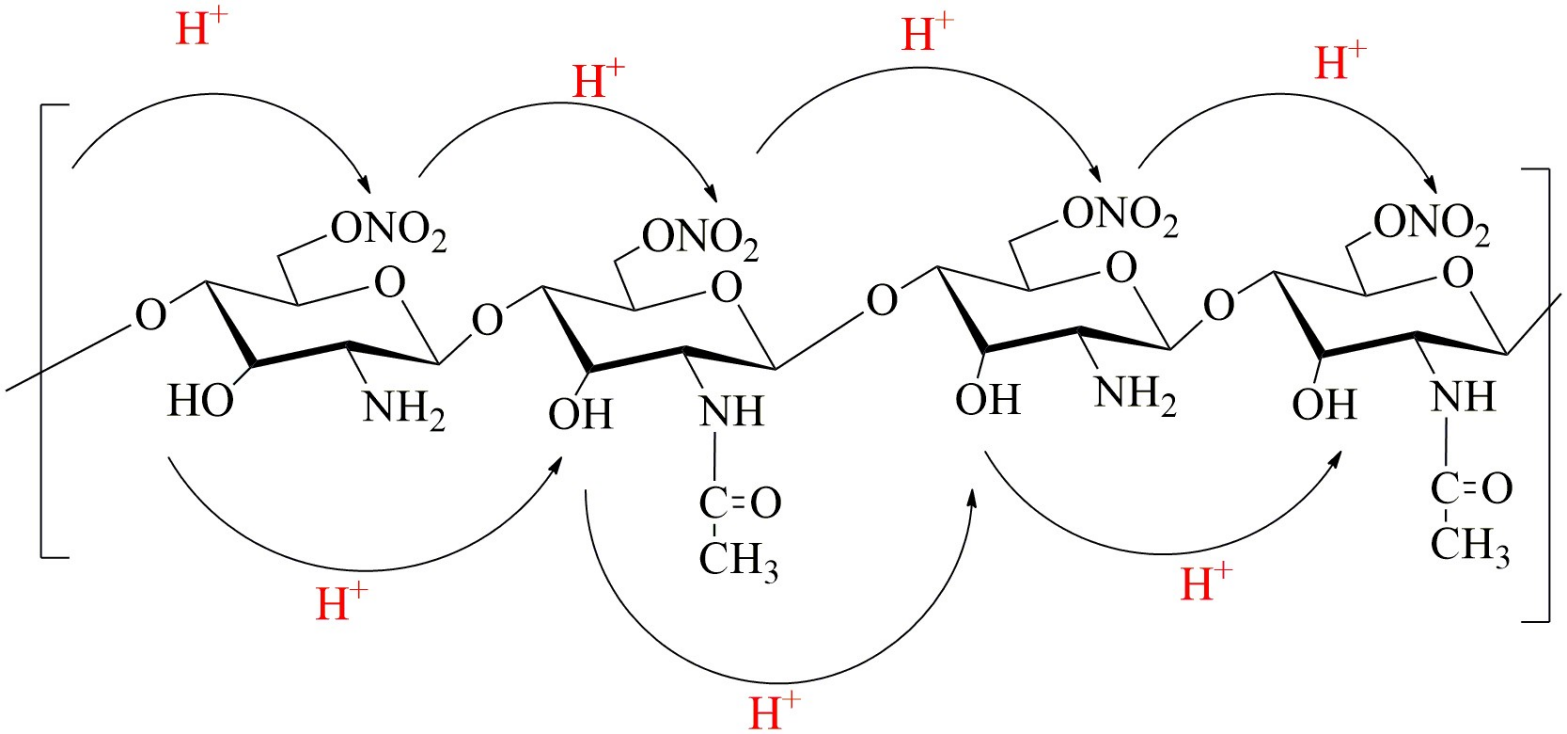
H^+^ conduction mechanism in O-nitrochitosan.

From Table 6, the conductivity of O-nitrochitosan polymer electrolyte membrane had achieved a higher value as compared to chitosan membrane. The conductivity increased about four orders of magnitude from 8.88 × 10^−10^ S cm^−1^ to 5.22 × 10^−6^ S cm^−1^. This showed that the chemical modification on chitosan by increasing the amount of oxygen in the structure of O-nitrochitosan has affected the conductivity properties of chitosan. The conductivity of blank O-nitrochitosan was expected to increase due to the oxygen atoms which possessed lone pairs of electrons that will coordinate with the cations. The more oxygen introduced to the chitosan structure, will mean that the number of active sites per repeating unit in the polymer also increased. This will enhance the migration of protons through the polymer matrix by providing more vacant sites for proton transport to occur. Since acetic acid was used in this blank system, H^+^ will dissociate from CH_3_COOH. Complexes of H^+^ from acetic acid will form with the oxygen atom from functional groups, O-H and O-NO_2_. The lone pairs of electron from O-NO_2_ were donated to H^+^ during complexation, as illustrated in Fig 9. When the electric field was subjected to the solid polymer electrolyte, the cations will transfer to the coordinating sites. It will transfer from one coordinated site to another in the same polymer chain or its neighbor due to the weak coordination of cations and the site along the polymer chain [32, 33]. The transportation of cations through the sites was also assisted by the segmental motion of polymer chain [13].

**Fig 10:**
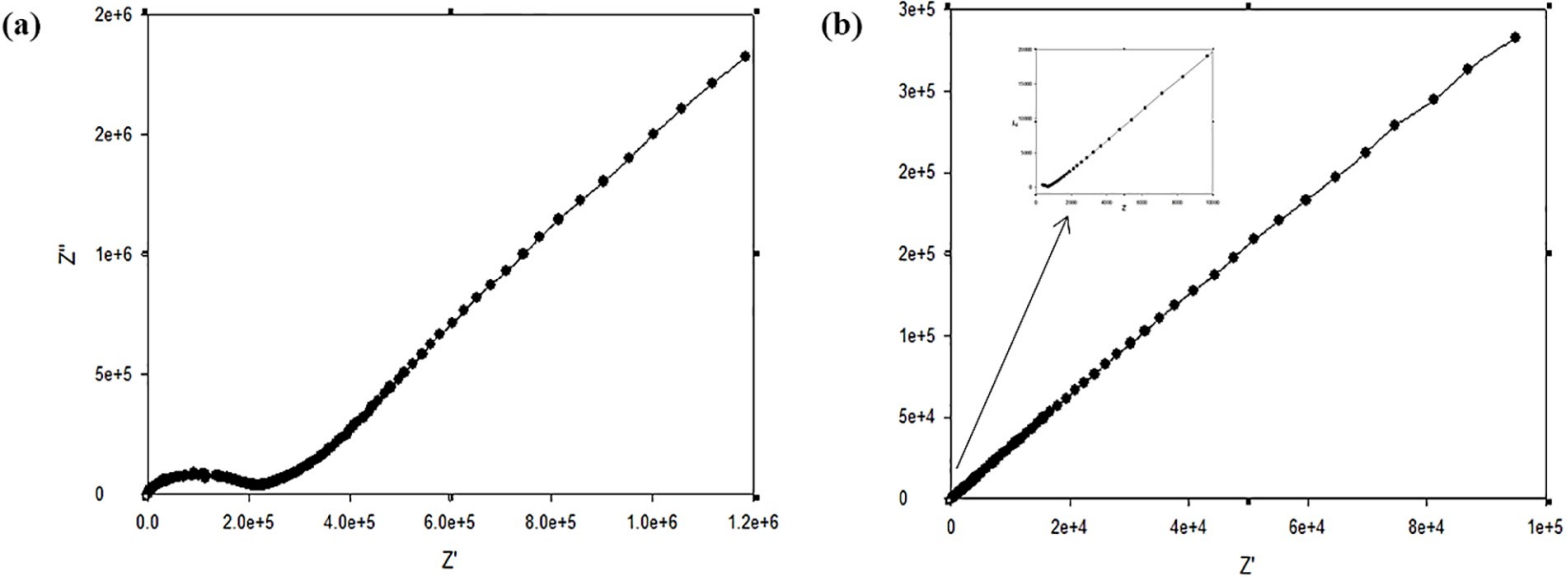
The complex impedances plot of (a) chitosan and (b) O-nitrochitosan. The data is available in S5 Fig.

Fig 10 shows complex impedance plots obtained on (a) chitosan and (b) O-nitrochitosan based green polymer electrolyte film. The plot was composed of two different regions, which were a slanted spike at low frequency and a semicircle at high frequency. The linear region was correlated with the existence of concentration gradients that gave rise to diffusion processes in the bulk electrolyte [34] while the semicircle part was linked to ionic conduction in the sample bulk resistance. The semicircle was embodied by a combination of resistor and capacitor in a parallel arrangement. The resistor can detect the ionic transportation within the polymer matrix while the capacitor measured the capacitance in the alternating field when immobile polymer chains became polarized [34]. In this system, the blocking electrode was used. So, the electrode/electrolyte interface can be reflected as capacitance. A slanted spike appeared instead of a vertical spike since the capacitance was non-ideal. The ideal capacitance will incline spike at 90°. The non-ideal spike will spike at an angle of less than 90°, due to non-homogeneity or roughness of the electrolyte/electrode interface. Besides, the degree of the slope and peak preference can be determined by relaxation time of mount up charge carrier at the interface [35].

#### XRD analysis of nitrochitosan films

**Fig 11:**
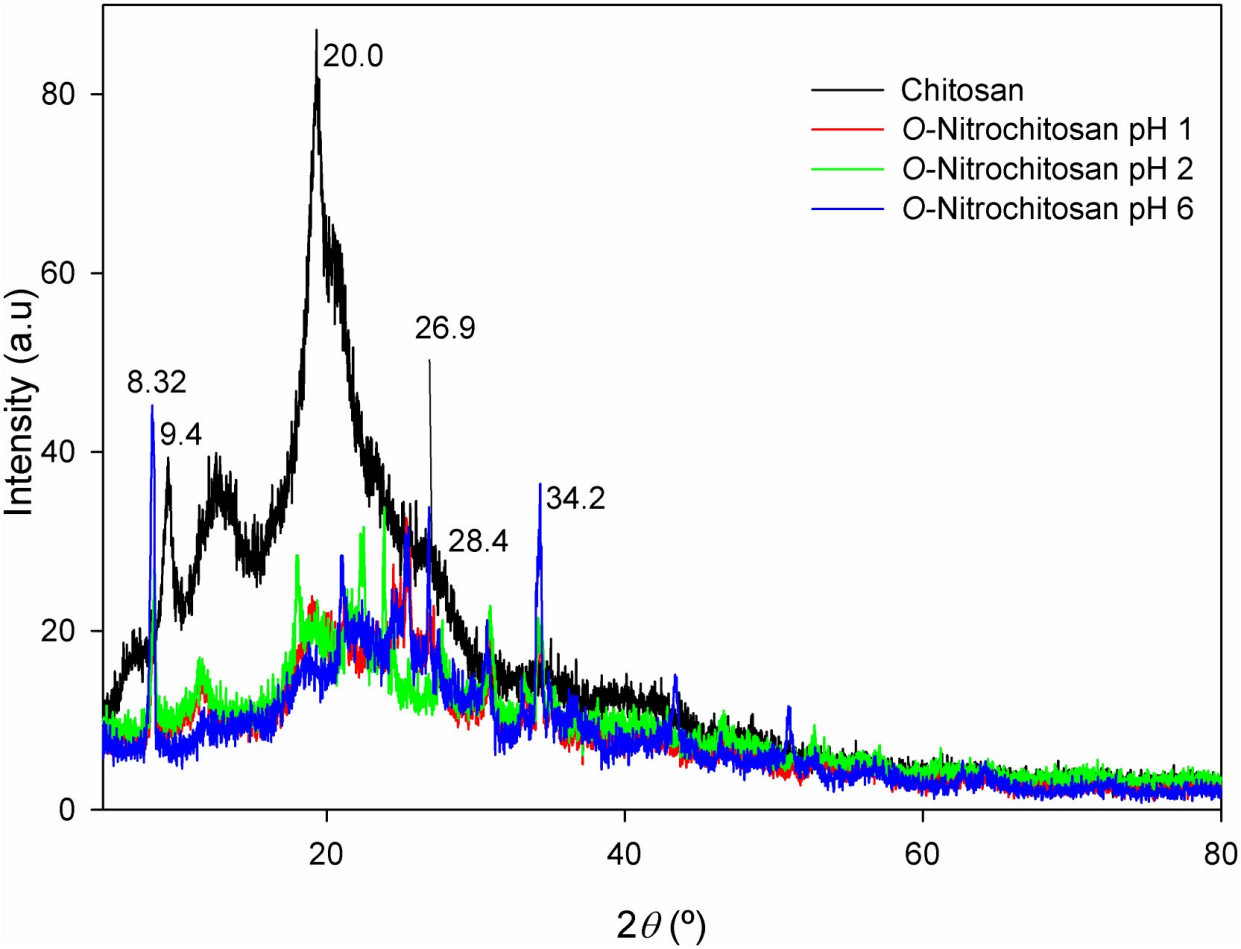
XRD diffractogram of chitosan film and its derivatives. The data is available in S6 Fig.

Fig 11 depicted the XRD patterns of chitosan film and its derivatives. The percentage of amorphous of all O-nitrochitosan films showed some increase when the powder was made into the film form. The crystalline peak of chitosan, which were around 2θ = 9.4°, 20.0° and 28.4°, were decreased because the hydrogen bond in the film was interrupted. The enhancement of the amorphous phase is important to increase the conductivity of the polymer. The polymeric chain will become more flexible in the amorphous phase, and thus increases the polymer segmental motion and will allow easier ion transport in this phase. The 2θ peak which, represent the crystallinity character of chitosan film forms and its derivatives are shows in Table 7.

**Table 7:** Peaks of 2θ in chitosan film and its derivatives.

Films	2θ in films
Chitosan	9.4°, 20.0° and 28.4°
*O*-nitrochitosan pH 1	8.32°, 25–26°, 30° and 34.35°
*O*-nitrochitosan pH 2	8.32°, 25–26°, 30° and 34.35°
*O*-nitrochitosan pH 6	8.32°, 25–26°, 30° and 34.35°

### Conclusion

The O-nitrochitosan in the acidic medium was successfully prepared and used as a host polymer for green polymer electrolyte. Chitosan-based electrolytes as a control experiment were also prepared. Both biopolymer electrolytes were prepared by solution casting technique. ATR-FTIR analysis confirmed that the interaction between the polymer host and acetic acid solvent has shifted the wavenumber of hydroxyl and amine band. The conductivity of the new chitosan derivatives showed its potential as green biopolymer electrolyte after their conductivity value showed some increases of about four orders of magnitude from ~10^−9^ to ~10^−6^ S cm^-1^.

## Supporting information

S1 Fig ATR-FTIR spectra of chitosan and its derivatives (powdered form) in acidic medium. (JNB)

S2 Fig XRD diffractogram of chitosan powder and its derivatives. (JNB)

S3 Fig TGA and DTG thermograms of chitosan and its derivatives. (JNB)

S4 Fig Second run thermal analysis curves for (a) chitosan, O-nitrochitosan pH 1, pH 2 and pH 6. (JNB)

S5 Fig The complex impedances plot of (a) chitosan and (b) O-nitrochitosan. (JNB)

S6 Fig XRD diffractogram of chitosan film and its derivatives. (JNB)
